# Optimizing Low-Cost Gas Analysis with a 3D Printed Column and MiCS-6814 Sensor for Volatile Compound Detection

**DOI:** 10.3390/s24206594

**Published:** 2024-10-13

**Authors:** Nela Skowronkova, Martin Adamek, Magdalena Zvonkova, Jiri Matyas, Anna Adamkova, Stepan Dlabaja, Martin Buran, Veronika Sevcikova, Jiri Mlcek, Zdenek Volek, Martina Cernekova

**Affiliations:** 1 Department of Food Analysis and Chemistry, Faculty of Technology, Tomas Bata University in Zlin, Vavreckova 5669, 760 01 Zlin, Czech Republic; n_skowronkova@utb.cz (N.S.); m1_zvonkova@utb.cz (M.Z.); aadamkova@utb.cz (A.A.); v2_sevcikova@utb.cz (V.S.); 2 Department of Automation and Control Engineering, Faculty of Applied Informatics, Tomas Bata University in Zlin, Nad Stranemi 4511, 760 05 Zlin, Czech Republic; m2adamek@utb.cz (M.A.); sdlabaja@utb.cz (S.D.); 3 Centre of Polymer Systems, University Institute, Tomas Bata University in Zlin, Trida Tomase Bati 5678, 760 01 Zlin, Czech Republic; matyas@utb.cz; 4 Department of Microelectronics, Faculty of Electrical Engineering and Communication, Brno University of Technology, Technicka 3058/10, 616 00 Brno, Czech Republic; martin.buran@vutbr.cz; 5 Department of Nutritional Physiology and Animal Product Quality, Institute of Animal Science, Pratelstvi 815, 104 00 Prague, Czech Republic; volek.zdenek@vuzv.cz; 6 Department of Microbiology, Nutrition and Dietetics, Faculty of Agrobiology, Food and Natural Resources, Czech University of Life Sciences Prague, 165 00 Prague, Czech Republic; 7 Department of Fat, Surfactant and Cosmetics Technology, Faculty of Technology, Tomas Bata University in Zlin, Vavreckova, 275, 760 01 Zlin, Czech Republic; cernekova@utb.cz

**Keywords:** chemiresistive gas sensors, 3D printing, sustainability, capillary, methanol detection, ethanol detection

## Abstract

This paper explores an application of 3D printing technology on the food industry. Since its inception in the 1980s, 3D printing has experienced a huge rise in popularity. This study uses cost-effective, flexible, and sustainable components that enable specific features of certain gas chromatography. This study aims to optimize the process of gas detection using a 3D printed separation column and the MiCS-6814 sensor. The principle of the entire device is based on the idea of utilizing a simple capillary chromatographic column manufactured by 3D printing for the separation of samples into components prior to their measurement using inexpensive chemiresistive sensors. An optimization of a system with a 3D printed PLA block containing a capillary, a mixing chamber, and a measuring chamber with a MiCS-6814 sensor was performed. The optimization distributed the sensor output signal in the time domain so that it was possible to distinguish the peak for the two most common alcohols, ethanol and methanol. The paper further describes some optimization types and their possibilities.

## 1. Introduction

The basics of 3D printing originated in the 1980s, and as all technical advances are driven by innovation, this promising approach has since found applications across numerous industries, showcasing significant potential for the development of various sectors. Currently, 3D printing is utilized across a wide range of sectors, including aerospace, automotive, healthcare, medicine, pharmacy, and consumer goods, underscoring its broad applicability and transformative impact [1,2]. The global 3D printing technology market is expected to grow significantly, rising from USD 12.6 billion in 2021 to an estimated USD 34.8 billion in 2026. This growth is possibly supported by the increasing adoption and advancements in 3D printing across various industries [3]. Unlike traditional manufacturing methods, 3D printing offers the ability to produce complex geometries with minimal material waste, which translates to lower production costs. These properties are very advantageous for use with gas sensors [4]. Beyond their affordability, these sensors offer advantages such as flexibility, easy adaptability, shorter manufacturing times, and relatively high measurement accuracy and reliability [5,6].

The utilization of 3D printing in the production of chromatographic columns has become particularly valuable, primarily because of the possibility to design the final product with the desired characteristics for its intended application [7].

The materials used for 3D printing also play an important role in shaping the final product properties [8], and they are constantly evolving. Polymers are generally the most widely used group of materials due to their strength, durability, hardness, and ability to withstand outside influences [9,10]. Based on the specific properties, a wide range of materials can be used to produce chromatographic columns. Three-dimensionally printed columns for gas chromatography (GC) have been mentioned in the literature, namely in studies by Phyo et al. [11] and Meng et al. [12]. For GC applications, both research teams used metals for the column manufacturing to withstand high temperatures during the analyses. The manufacturing of 3D printed columns has also been employed in liquid chromatography (LC), and in the future, it might become a widespread technique for both preparative and analytical columns in both research and industrial applications [13]. For high-performance LC applications, materials able to withstand high pressure must be selected, such as silica, hydroxyapatite, acrylates, agarose, and cellulose [14].

This article presents some aspects and findings of measurements with a 3D printed column manufactured using the Fused Deposition Modeling (FDM) method and with a MiCS-6814 sensor used as a detector to enable the improved design and optimization of similar devices for the increased resolution and detection of food samples. The main goals of this study were to optimize the gas detection process using a 3D printed separation column and the MiCS-6814 sensor and to enhance the ability to differentiate individual samples. Although this study did not primarily aim to analyze individual gas components in samples in a way to compete with the much more complex and expensive capillary gas chromatography methods, these methods and the presented experimental setup share the same working mechanism. Therefore, principles of gas chromatography were adopted by the authors and modified to fit the device consisting of the 3D printed components and chemiresistive sensors.

Besides the optimization of the device, the goal was to maximize the sustainable properties of the equipment by keeping material and energy requirements to a minimum. This also contributes to reducing economic costs.

## 2. Materials and Methods

### 2.1. Samples and Auxiliary Materials for Experiments

This article is based on the idea of increasing the selectivity of recognizing food samples and their condition. Since the chosen chemiresistive sensor MiCS-6814 is sensitive to basic simple compounds (especially alcohols), the following substances were used in the experiments:

- Methanol—99.8% Methanol G.R., Lach-Ner, s.r.o., Neratovice, Czech Republic, CAS: 67-56-1, EINECS: 200-659-6;
- Ethanol—96% Ethanol, Ing. Petr Švec-PENTA s.r.o., Prague, Czech Republic, CAS: 64-17-5, EINECS: 200-578-6;
- Toluene—99% Toluene G.R., Lach-Ner, s.r.o., Neratovice, Czech Republic, CAS: 108-88-3, EINECS: 203-625-9.

Additionally, commonly available beverages and other chemicals were used in this study:

- Drink “Vodka”—Vodka, GAS Familia, s.r.o., Stará Ľubovňa, Slovakia, alcohol content of 37.5% vol. Composition of the drink: very fine refined spirit of high quality and demineralized water (expected composition is 62.5% vol. water and 37.5% vol. ethanol).
- Drink “Tuzemsky”—Tuzemsky, GAS Familia, s.r.o., Stará Ľubovňa, Slovakia, alcohol content of 37.5% vol. Composition of the drink: ethanol, aroma, sugar, coloring, and plain caramel color.
- Gas from a cigarette lighter—Royce cigarette lighter (commonly reported as butane (97.5%) and propane (1.2%)).

As the carrier gas, normal room air filtered through a NY 0.22 µm syringe filter (Chromservis s.r.o., Prague, Czech Republic) was used.

### 2.2. Experimental Equipment

This study was focused on the optimization aspects of the device—an experimental electronic nose. The fundamental concept is outlined in our previous work [15], and the device is described in detail in the article by Zvonková et al. [16]. The specific modifications of the device and measurement methodology, compared to these earlier studies, against that article are described in the section below, and in Results and Discussion section presents the descriptions of individual optimizations.

**Figure 1:**
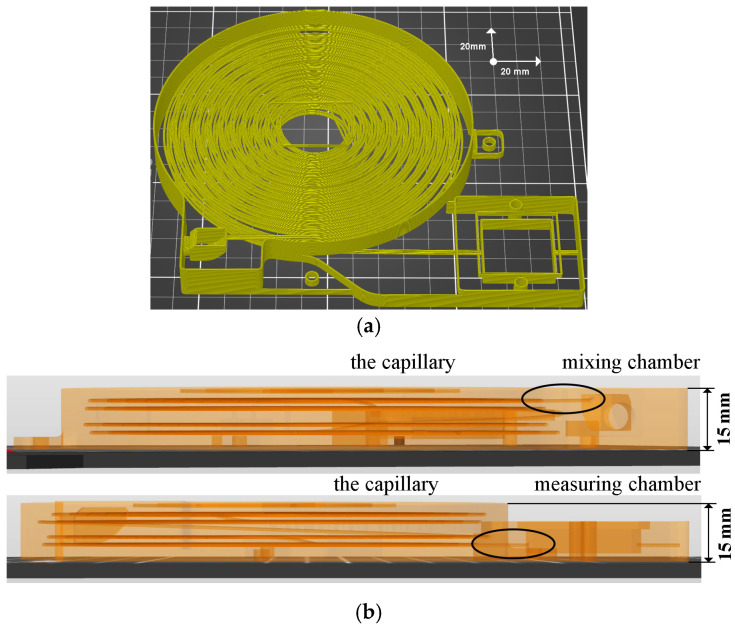
Views of the PLA capillary block model in Prusa Slicer 2.7.4+win64 software (Prusa Research a.s., Prague, Czech Republic). (**a**) General view of the capillary block model. (**b**) Side views of the capillary block model showing the mixing chamber, measuring chamber, and four-layer capillary and their connections.

A 3D printed PLA capillary block with a mixing and measuring chamber (Figure 1) was used as the basic part of the experimental device. Compared to the previous version described by Zvonková et al. [16], the block was increased to four layers of capillary threads, and the capillary length was extended to 9.4 m. The diameter of the capillary was increased to 0.9 mm. To prevent the leakage of carrier gas and sample, only one single hole was created at the entrance to the mixing chamber, and the chamber volume was reduced to approximately 0.8 mL. The other parameters remained unchanged.

Once the sample, driven by the carrier gas from the syringes, passes through the separation capillary, its individual components are measured using the chemiresistive MiCS-6814 sensor (SGX Sensortech, Neuchâtel, Switzerland) [17]. The sensor is then connected to an ESP-WROM-32 microcontroller (Espressif Systems, Shanghai, China) on the Wemos LoLin 32 ESP-WROOM-32 board, which controls the experimental measuring device and transmits the measured data to a PC-class computer. A more detailed description of the equipment, data processing, and their evaluation is given in the article by Zvonková et al. [16]. For the data processing and graphical representation of the measurement outcomes, Microsoft® Excel® 2019 (Microsoft Office Professional Plus 2019, Microsoft Corporation, Redmond, WA, USA) was used. The optimization of some device parameters and the optimization of data processing are described below.

The measuring device itself consists of several interconnected parts. The basic element is a compact 3D printed PLA unit containing a capillary for separating the mixture’s components, a mixing chamber for mixing the gas sample with the carrier gas, and a measuring chamber equipped with a chemiresistive sensor. The apparatus further comprises a sample dispenser and a carrier gas dispenser driven by a gear motor. Everything is controlled and monitored by a measuring and control unit controlled by an ESP-32 microcontroller. The main part of the device is a compact block with a capillary. Emphasis was placed on the proper integration of the mixing and measuring chamber into a compact unit with a capillary.

The gas sample was collected at room temperature at a pre-determined height, precisely just above the surface of these volatile compounds, using a syringe needle, with the beveled tip of the needle in contact with the liquid while ensuring that the lumen remained out of contact with the liquid.

## 3. Introduction to Optimization and Measurement with Experimental Equipment

The optimization of the experimental equipment is based on the measured results, practical experience gained during measurements with the experimental system and its previous versions, and knowledge from the literature in the field of capillary gas chromatography. The optimization to obtain the best measured signal can be broadly divided into two groups: experimental equipment-related optimizations and measurement-related optimizations.

**Figure 2:**
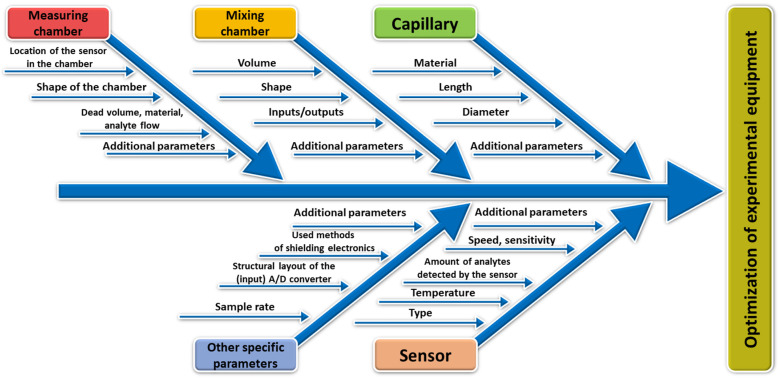
A simple Ishikawa diagram of the optimization of the experimental equipment.

For a particular experimental equipment, the device-related group can be further divided into the following parts (Figure 2):

- Capillary—material, length, diameter, cross-sectional shape, capillary shape, capillary internal surface properties (so-called stationary phase type), etc.;
- Sensor (detector)—type, the number of types of analytes that the sensor detects, sensitivity, speed, temperature, etc.;
- Measuring chamber—location of the sensor in the chamber, shape of the chamber, dead volume, material, analyte flow, location, and size of the inlet/outlet, etc.;
- Mixing chamber—volume, shape, inlet/outlet;
- Other specific parameters and characteristics of the experimental apparatus.

The measurement-related group is further divided into the optimization of the following:

- Sample—volume, concentration, time and rate of dosing, sample preparation and collection, etc.;
- Carrier gas—gas type, flow rate, purity, etc.;
- Temperature—Temperature of the various essential parts of the equipment;
- Analysis time—duration of measurement, optimization for peak resolution, etc.;
- Calibration—selection of standards used for qualitative and quantitative analysis;
- Result processing—selection of the order of mathematical operations to be performed on the measured raw data;
- Other specific parameters and properties of the measurement.

Some of the parameters known from classical gas chromatography lose their significance with the equipment used. An example is the “Split ratio” parameter, which indicates the ratio of the sample entering the column to the sample leaving the column.

Although modern chemiresistive sensors can achieve even lower detection limits than typical detection systems used in gas chromatography, their main disadvantage is the large time delay. This limits the speed of evaluation of changes in analyte concentration in conventional commercial time-domain sensors (as will be shown below) and their deployment in faster systems, such as gas chromatography where detectors with faster time response must be deployed.

## 4. Results and Discussion

### 4.1. Selected Device Optimization Processes

The optimization of the experimental equipment used was mainly focused on the capillary block with a mixing and measuring chamber. The capillary was changed from the previous version with two layers of threads, a length of approximately 4.7 m, and diameter of 0.7 mm, as described in our earlier publication [16], to a capillary with four layers of threads, approximately twice the length at 9.4 m, and a diameter of 0.9 mm. This modification has reduced the risk of the capillary collapsing and clogging during manufacture and increased the absorption surface of the capillary by increasing its diameter. However, it also increases the possibility of the sample passing only through the middle of the capillary without interacting with the capillary walls. A more detailed description of the capillary block is given in Section 2.

Further optimization of the capillary block was mainly focused on optimizing the shape of the measuring chamber with the following goals:

- Minimizing the dead space (to prevent analyzed gas from becoming trapped in the measuring chamber and causing measurement errors).
- Creating a path for the gas to pass through the chamber (to bring the analyte from the capillary to the sensor quickly and to drain it sufficiently fast).
- Sealing the chamber properly.

Another important parameter is the placement of the sensor in the measuring chamber (the sensor should be in the center of the chamber and as close as possible to the capillary outlet). However, this parameter has remained unchanged due to the use of the original printed circuit boards with sensors.

**Figure 3:**
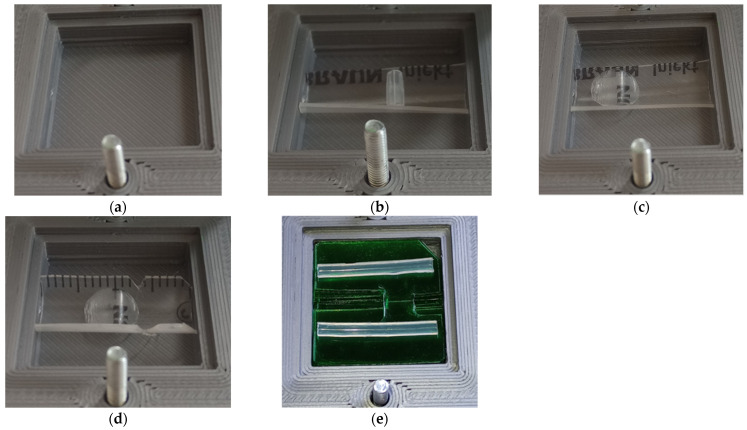
Modifications and improvements of the measuring chamber (internal square dimension 27 × 27 × 4 mm): (**a**) an empty chamber; (**b**) a canal chamber with baffle; (**c**) a chamber with a channel and its constriction in the sensor area; (**d**) a chamber with a channel, narrowing in the sensor area, and cutouts to improve analyte drainage; (**e**) a layered green lined chamber with a molded channel and rubber leak guard.

Figure 3 shows several configurations of the chamber that were tested to satisfy these requirements:

1. Clean chamber (Figure 3a)—default empty version.
2. Chamber with U-channel and baffle (Figure 3b)—the U-channel was made by cutting out the wall of a 5 mL syringe. The baffle was made from a rubber tube and placed in the gas path upstream of the sensor. Even though the U channel was well flushed with the sensor circuit board, there was still a lot of dead space in the chamber. In addition, the U-channel was slightly deformed (found after opening the chamber).
3. Chamber with a U-shaped channel and narrowing in the sensor area (Figure 3c)—a taper was inserted into the U-channel to direct the flow of analyte into the sensor (to the sensor elements through the protective metal grid) and back again. However, in practical measurements, the response was reduced—presumably, the metal grid of the sensor was clogged by the created constriction (the sensor was probably too small and the constriction too large).
4. U-channel chamber with a taper in the sensor area and cutouts to improve analyte drainage (Figure 3d)—similar to the previous version with notches in the U-channel wall for faster analyte drainage from the sensor. The narrowing is shifted behind the sensor.
5. A chamber with a layered green liner with a shaped channel containing rubber leak guard (Figure 3e)—currently, the final version has a channel shaped from individual layers of smooth PET film stacked on top of each other and two rubber bands sealing the channel area from the rest of the compartment.

**Figure 4:**
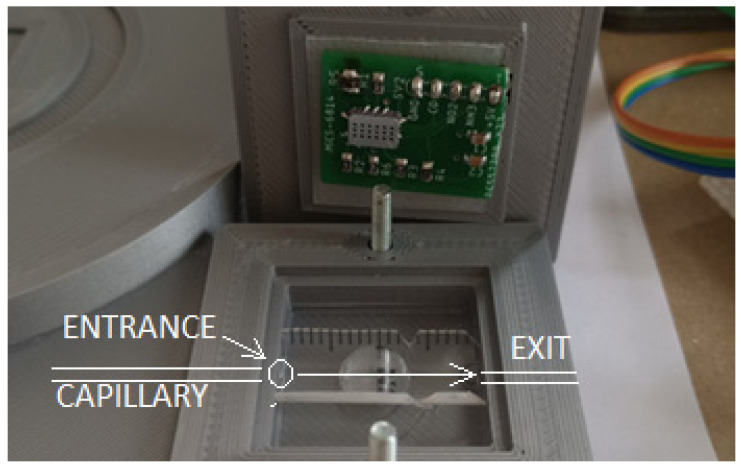
Overall positions of the measuring chamber and the sensor plate. The entrance to the chamber is on the left from the capillary, on the right the exit to the open space.

The overall position of the measuring chamber and sensor plate is shown in Figure 4. The entrance to the chamber from the capillary is on the left side of the chamber in the middle, and the exit from the chamber to the open space is on the right side of the chamber in the middle.

**Figure 5:**
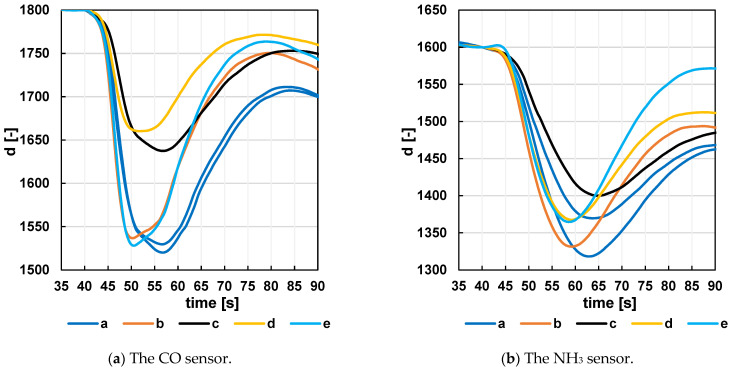
Response of CO (**a**) and NH_3_ (**b**) sensor to a 1 mL Vodka sample for different measuring chamber configurations. The labeling of the curves (letters a–e) is identical to the labeling of the measuring chamber configurations in Figure 3. The data were preprocessed before standardization.

Tests for each configuration were performed on a 1 mL vodka sample, and the goal was to detect a reaction characterized by a high but narrow peak. Due to time constrictions, each test was performed only once, with the exception of configuration (a), which was tested twice. Despite this limitation, some observations can be drawn from the CO sensor results shown in Figure 5, allowing us to derive some hypotheses. In the empty chamber without an insert, the sensor response to alcohol is indeed high, but the peak is very broad. It is assumed that the analyte remains in the chamber (due to the large dead space volume) and has a prolonged effect on the sensor.

For configurations (c) and (d) with a narrowing in the sensor area, the required airflow through the protective grid to and from the sensor likely did not occur, leading to restricted flow. This resulted in a reduction in the signal. Configuration (b), with a U-channel and a baffle behind the sensor, appears to be one of the best configurations. However, with this configuration, there was a risk of channel wall deformation followed by gas leakage into the unfilled area of the chamber. Therefore, configuration (e), with the insert composed of layered PET film, was chosen as the best configuration. Each layer is made with a defined cut-out groove structure. When these films are stacked on top of each other, a channel with a defined cross-section at each location is formed. The advantage of this design is the almost complete filling of the dead space (outside the actual channel and the cut-out for handling) at the bottom of the chamber. A sensor circuit board is located at the top of the chamber. In addition to the sensor, other components and a connector are on this board, which also create dead space between them. The sensor and channel area are separated from this space by two U-shaped rubber bands (sealing between the circuit board and the cut-shaped foil).

The graph for the NH_3_ sensor shows a slower response of this sensor compared to the graph for the CO sensor. Although the response for configuration (e) with the layered foil insert is not the highest, the peak appears to be narrower compared to the others. Obtaining data that can subsequently be characterized straightforwardly based on clear visible peaks is one of the essential steps for optimizing measurements with the proposed system.

**Figure 6:**
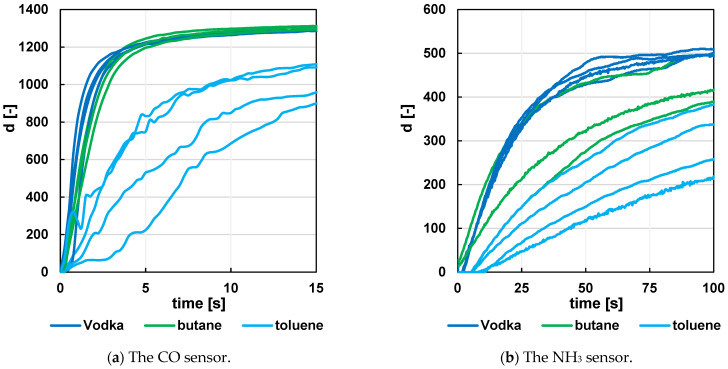
Time response of the sensor (raw data from the A/D converter) to the departure of the analyte Vodka, butane, and toluene from the sensor area. The starting time point is 0 s—the first recorded signal rise at the CO sensor.

**Figure 7:**
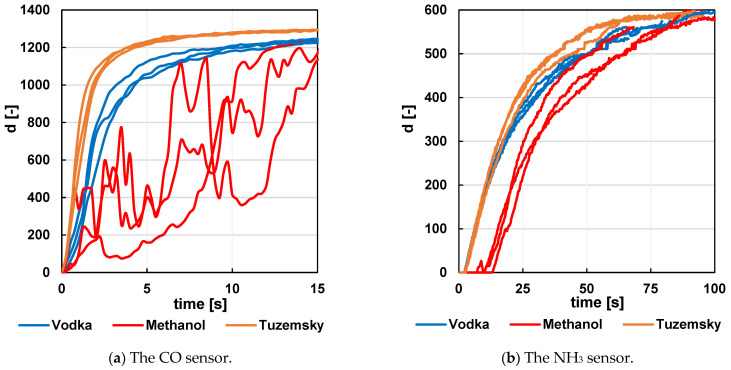
Time response of the sensor (raw data from the A/D converter) to the departure of the analyte Vodka, methanol, and Tuzemsky from the sensor area. The starting time point is 0 s—the first recorded signal rise at the CO sensor.

Not only is the rapid removal of the analyte from the measuring chamber important in this optimization, but the velocity characteristics of the sensor itself must also be taken into account. A simple experiment was conducted with the sensor placed in a holder just above the test tube with the measured solution, and its response was monitored when the sensor was moved away into free space. The experiment showed that molecules of some analytes left the sensor faster than molecules of other analytes (Figure 6 and Figure 7). This may cause undesirable interaction between the individual recorded peaks and potentially lead to their misinterpretation. For this reason, this type of detector is particularly suited for analytes with components characterized by a sufficiently rapid exit from the sensor.

The curve areas of each analyte can be traced from the figures. Although it is suggested that analytes with smaller molecular weights bind to and leave the sensor with a shorter time constant than those with larger molecular weights, the methanol curves show that other aspects will also affect the time constant.

Several sensors were used for the measurements, which may not have had identical characteristics or resistor network settings. For this reason, the same samples in Figure 6 and Figure 7 do not exhibit exactly the same characteristics.

During this optimization, two additional minor experiments were conducted. The first involved adding a thin polyethylene film between the sample syringe and the injection needle. This film prevents leakage of the sample from the syringe through the needle’s tip before the moment of sample dispensing, such as during the handling of the syringe or with more volatile samples. When pressure is applied to the plunger, the thin film bursts and the sample is injected into the system at the exact moment of dispensing. This ensures that the signal reduction visible at the beginning of the measurement is only a reaction of the sensor to the passage of the carrier gas, and not to a leaked part of the sample into the system before dosing. The downside of this solution is the need to insert a foil between the syringe and the needle after the sample is drawn into the syringe. This may cause a time delay during which a small portion of the sample may leak out of the syringe. Therefore, a larger volume of the sample is drawn into the syringe. With the needle and foil slightly loosen, the excess volume is expelled from the syringe, followed by immediate needle-to-syringe contact. Another disadvantage is that the foil may not burst when the sample volume is small. With larger sample volumes, the film may burst, but the sudden pressure change inside the system may affect the sensor signal.

The second minor experiment involved changing the flow rate by adjusting the voltage to the thruster motor for the carrier gas from 12 V to 5 V. This optimization will be described in more detail below.

Other optimizations that can be included among other specific parameters and features of the experimental setup include increasing the sampling speed compared to the older version of the system, from one sample to four samples per second. This sampling rate allowed for a more accurate signal characterization and improved signal processing.

It is also important to mention one negative feature of the device that caused a significant delay in some measurements before it was identified. In a certain way, this problem can be considered an optimization. Specifically, the SD card used for data recording must have sufficient capacity. Initially, an older SD card with a capacity of 64 MB was used for data recording. During measurements with this card, it was discovered that significant noise was introduced into the data if the file exceeded half the card’s capacity. This issue resulted in significant delays in the work, as the connection between the increased noise in the measured signal and the capacity of the SD card was not recognized for some time.

### 4.2. Selected Measurement Optimization Processes

The measurement usually consisted of the following basic steps:

1. Setting the data acquisition program and mechanism to the pre-start position.
2. Manually flushing the capillary with clean air from a 165 mL syringe (often several times).
3. Inserting a syringe containing a defined volume of clean air into the device.
4. Adjusting the carrier gas mechanism to the start position with the carrier gas pusher mechanism against the syringe plunger (eliminating dead time).
5. Removing the syringe sample and inserting it into the dispensing mechanism.
6. Starting the measurement (0 s)—measuring without activating the motors to establish the system baseline.
7. Automatic start of the push mechanism motors (10 s).
8. Automatic sample pitch (40 s).
9. Self-measurement.
10. Manually terminating the measurement (often after the carrier gas dose in the syringe is depleted).
11. Returning the pusher mechanism to the pre-start position.
12. Manually flushing the capillary with clean air from the 165 mL syringe to remove any sample residue (often several times, often combined with step 2 before the next measurement).
13. Data recording, storage, and processing.

**Figure 8:**
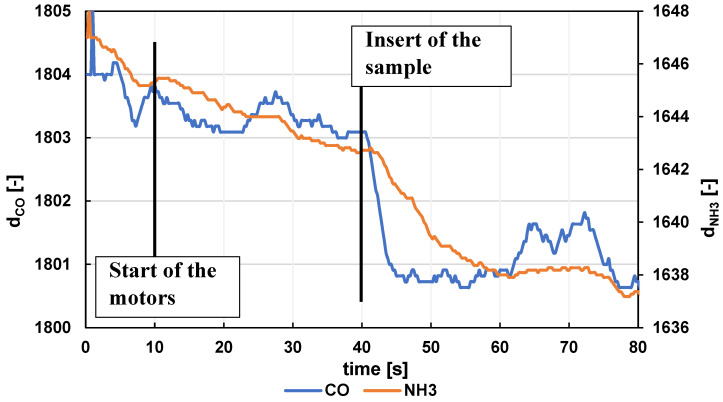
Example of the start of a measurement with a clean air sample (values after a centered moving averaging m = 11).

An example of recording the start of a measurement after a centered moving aver-aging is shown in Figure 8.

Some of the basic parameters were not optimized because they were directly determined by the work’s objective, the device’s capabilities, or because changing them would have been too difficult. The device was designed with an emphasis on minimizing cost, reducing waste and ensuring high sustainability. These parameters include the following:

- Carrier gas:
  - Type of carrier gas—air (determined by minimum cost);
  - Purity of carrier gas—determined by the simple filter used in line with the minimum cost.
- Temperature—the temperature of the individual basic components of the device was kept at room temperature because changing and controlling it would make the experimental device more expensive. In addition, the column block material could not be heated to the temperature typically used for gas chromatographs (generally around 200–250 °C).

Other basic parameters could only be selected, adjusted, or modified to a limited extent, particularly the following:

- Calibration standards—These are determined by the substances to which the exact type of sensor responds, as specified by the manufacturer. In our case, ethanol, food-grade methanol, and toluene were used as calibration standards (see the section on materials used). In small-scale production or even domestic settings, this selection is limited. One of the most readily available standards for qualitative analysis appears to be an alcoholic disinfectant with an ethanol content of up to 90%, or an alcoholic beverage such as vodka, which typically contains around 38% ethanol. Another readily available solution is spirit vinegar, with an acetic acid content of approximately 8%. However, the sensor data sheet (MiCS-6814) does not specify its response to the vapors of this solution; moreover, the solution may contain other additives to which the sensor could react. In addition, other substances can be used as calibration standards and can be purchased in the general commerce. However, their sale may be restricted (e.g., for toluene diluent), or their exact concentration and composition may be unknown (e.g., butane from a cigarette lighter, CO2 produced by a siphon, or propane–butane from camping fuel canisters). Other gasses, such as helium for filling children’s balloons, can be commonly purchased, but the sensor used must be sensitive to them. Under standard laboratory conditions, it is then possible to work with other calibration standards.
- Sample volume and dosage—A syringe with a 2 mL capacity was selected for sample injection. This syringe is a commonly available small-volume model compatible with a standard needle providing a sufficiently long plunger to facilitate dispensing within an accessible system. The standard volume used in this research was 1 mL or 2 × 0.7 mL. Previously, the full range allowed by the dispensing device (approximately 1.5 mL) was utilized. However, as mentioned above, dosing affects the pressure in the measuring system, to which the sensor is sensitive. Therefore, it is anticipated that a larger analyte dose volume will result in more significant pressure changes and, consequently, greater interference in the signals detected by the system. On the other hand, a smaller dose (below 0.5 mL) may be insufficient to break through the separating thin polyethylene film and penetrate the measuring system at the appropriate time.

Furthermore, the volume of the mixing chamber was calculated to be 0.81 mL. It is assumed that the volume of the sample to be dosed should not significantly exceed the volume of this chamber and its supply path (including the needle and syringe outlet volumes). This total volume was estimated to be 1mL. The recommended sample volume in the current system is between 0.5 and 1 mL for a single sample.

For sample preparation, the individual components of the resulting mixture of pure samples of calibration materials (see the list of materials used) were initially drawn into separate syringes and then combined in a mixing vessel. A sample was subsequently taken from this container for measurement. However, the procedure of mixing the sample from the two syringes with clean calibration samples directly in the mixing chamber before entering the capillary proved to be a more advantageous procedure at the conclusion of these optimizations. It is assumed that this approach resulted in less leakage. The disadvantages included limitations in the number and volume of samples, as well as increased syringe and needle losses. For this reason, a total sample volume of 1.4 mL was selected (with the same sample ratio, a volume of 0.7 mL per syringe).

**Figure 9:**
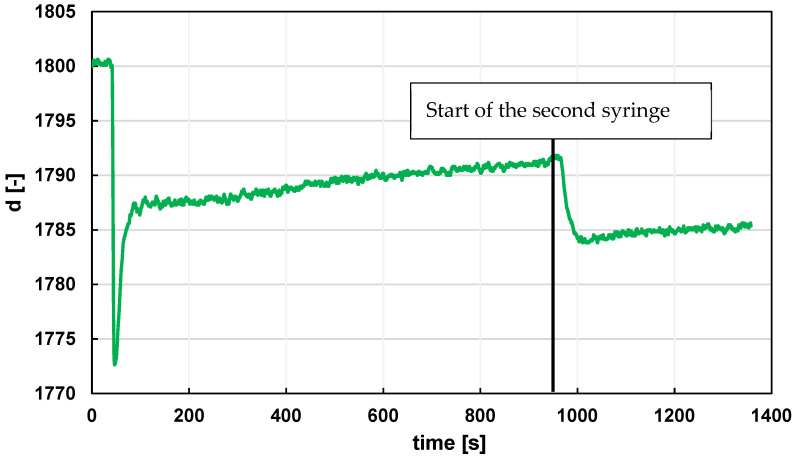
Example of CO sensor response when a second syringe is connected, and the flow rate is changed from 0.0177 mL/s to 0.0830 mL/s for a 1 mL sample of a 1:1 mixture of natural gas (methane) and food grade ethanol. The data were preprocessed before standardization.

- Carrier gas flow rate—It has a direct impact on the duration of the measurement. It is one of the limiting factors for this device; a minor trade-off is that the carrier gas can be repeatedly obtained for free by manually drawing it into the syringe without the use of additional energy sources and materials. Since the carrier gas is obtained from a syringe of a certain volume, measurements can only be performed until that volume is depleted. Thus, the higher the flow rate, the shorter the measurement time. However, the device is equipped with the capability to accommodate two syringes, allowing for the combination of their volumes. Alternatively, it is possible to use a slow flow rate during the initial phase of the measurement and a higher flow rate during the later phase.
  In practice, it was possible to vary the flow rate of the gas as follows:
  ○ By adjusting the gearbox as follows:
    ▪ Quick gear—originally designed to return the mechanism to its base position. However, due to the impracticality of constantly loosening and tightening the screws, this idea was abandoned. During test measurements in the standard configuration with the motor set to 12 V, the system pressure was already so high that the teeth on the motor axle began to skip, threatening to destroy the gearbox. When the voltage was reduced to 6 V on the motor, the measurement took about 240 s. In this configuration, using two 165 mL syringes, the carrier gas flow rate was approximately 330 mL over 240 s, which equates to s = 1.375 mL/s.
    ▪ Slow gear—currently used for both measuring and returning the mechanism to its base position.
  ○ Voltage to the pushing mechanism motor—The voltage to the motor is primarily drawn from an external adjustable source (0–20 V) and can be varied from 5 V to 12 V for measurement purposes. At lower voltages (bellow 5 V), current is supplied directly from the microcomputer board and USB port, which poses a risk of overloading the microcomputer’s power supply. The operating voltage at the push–pull motor of the feed motor is specified by the manufacturer at 12 V. It can be temporarily increased to 13 V for short periods. Further motor control (e.g., by PWM modulation) has not yet been tested.
- Syringe volume change—For measurement, syringes with standard volume 20 mL (maximum volume 24 mL), 50 mL (maximum volume 60 mL), and 150 mL (maximum volume 165 mL) were used. The maximum volumes were utilized for the measurements. Syringes with a small volume but longer length are advantageous for achieving low flow rates. Furthermore, a dual syringe holder provides additional flexibility by combining different syringe sizes. This is particularly useful, for example, for increasing the flow rate at the end of the measurement and for capillary flushing (enabling signal control and time saving). The potential disadvantage is the sensor’s reaction to a change in flow rate (see Figure 9).

The limits for the carrier gas flow rate for different system configurations are shown in Table 1.

**Figure 10:**
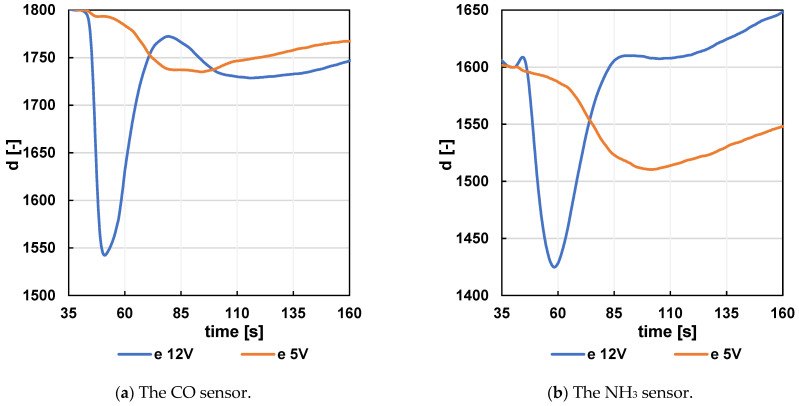
Response of the CO (**a**) and NH_3_ (**b**) sensor to a 1 mL Vodka sample at different motor voltages. The data were preprocessed before standardization.

As an example, the change in sensor output response when the voltage on the carrier gas pusher motor is changed from 12 V to 5 V in configuration (e) was compared. The configuration designation (e) is the same as the measurement chamber configuration designation in Figure 3. The change in sensor response is shown in Figure 10. The figure shows a significant distribution of the signal over the time, as well as a substantial reduction in signal strength.

The voltage on the dispenser motor is the same as the voltage on the main motor of the pusher mechanism. The voltage ranges from 5 to 12V. For low voltages, however, the motor often cannot overcome the force caused by separating polyethylene film, so it is necessary to assist the motor by hand. Due to this, the moment of sample entry into the system can range from 40 to 42.5 s.

**Figure 11:**
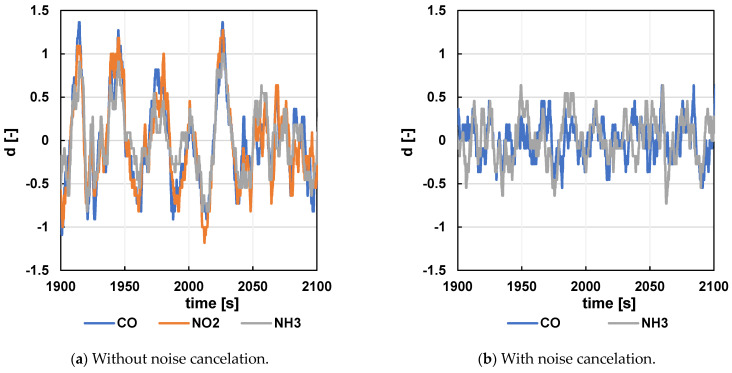
Noise elimination using the NO_2_ sensor signal.

One of the optimizations entirely under the control of the measuring equipment operator is the processing of the measured signal and subsequent result analysis. This mainly involves the selection of the order of mathematical operations performed on the measured raw data. In this area, two optimizations were implemented in this study compared to those in the previous version:

- Use of NO_2_ sensor signal for noise elimination—The NO_2_ sensor is mainly unresponsive to food samples at low concentrations (it primarily responds to NO and NO_2_) but significantly reflects the same noise as the other two sensors, as shown in Figure 11 below (e.g., at time 2025 s). Further calculation is than performed. For example, to eliminate the noise from the CO sensor signal, the following equation is used:

CO_eliminated_ = CO_measured_ − NO_2measured_ + NO_2average_ (1)

In the case of a significant response of the NO_2_ sensor to the given analyte, this calculation cannot be used at all. In the case of a moderate response, the average NO_2_ value is substituted in the equation with a value calculated from the trend in the curve.

**Figure 12:**
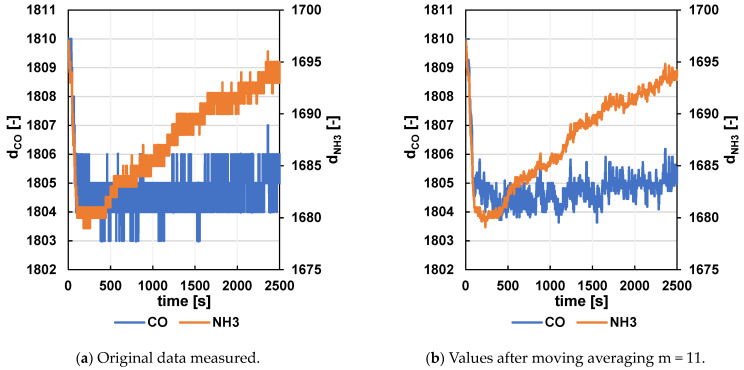
Example of the whole measurement process with air.

- Signal averaging—After noise elimination, centered moving averaging is used, most often with parameter m = 11; see Figure 12. This parameter was also used in previous circuits. Since the sampling rate in this version was four times slower, the averaging covered a section four times longer. Therefore, parameters similar to the earlier time span (m = 41 or m = 45) were also tested, as well as the left moving average, to avoid taking into account future samples that will be measured after the current count. However, this approach could potentially result in the loss of some detail or cause an undesirable time shift in the final characteristic.

The selected device optimizations discussed in Section 4.1, can be further improved by incorporating methods such as “pre-separation”, which involves the use of specific filtration components or the functionalization of the column shape and surface. This modification has been demonstrated to contribute to the differentiation of methanol and ethanol peaks in the final graph [15,18].

This process is essential not only for identifying differences in sensitivity levels in the final graph, but also for accurately detecting specific peaks corresponding to different masses of substances, especially in mixtures containing both methanol and ethanol. Although gas chromatography remains the most reliable method for this type of analysis, the possibility of improving alternative methods is constantly being explored. Additionally, the use of multiple sensors, both inlet and outlet, could be advantageous in obtaining more accurate data. Using an array of sensors in conjunction with advanced data processing techniques could achieve a more comprehensive and detailed result [19].

Due to the device’s operating principle and the “low-cost” philosophy, the use of gas as a pre-treatment method is understandably not feasible [20].

Automation can also affect the actual insertion of samples. Low-cost sample dispensing functions have also been developed and should be included in the next version of the device. In addition, an automatic air pump without volume limitation could also be beneficial for experimental continuity and improving measurement accuracy [21,22].

The sensitive parts of the sensors have also been modified in other studies using nanofabrication methods. Combined with machine learning techniques based on conductivity level recognition, this method has been proven to improve methanol and ethanol recognition, but it also provides a way forward for similar devices in the future [23].

Potential improvements to the injection system should include a stable gas source for mixing the gas to be analyzed. Syringes with limited gas capacity were used for the present study. However, instability in the curve of the graph was measured between injections from the first and second syringes. The solution to this point is a pressure cylinder equipped with a manometer to determine the specific working pressure. Applying a higher pressure level may be applied for flushing. Typical gasses used in the food industry, such as nitrogen and carbon dioxide, are appropriate for this purpose as they should not affect the properties of the measured sample [24].

### 4.3. Results Achieved with the Optimized System

After the optimizations were performed, an ethanol and methanol resolution test was conducted at the end of the work. An additional needle and syringe for the analyte were added to the mixing chamber, and the two analytes were mixed in their pure forms. Dispensing was carried out manually due to there being only one dispensing device for the sample. One syringe with a total volume of 60 mL was selected as the source of carrier gas, with a flow rate of 0.0333 mL/s, and a voltage of 5 V was set for the motor of the pusher mechanism. The following combinations were chosen for the measurements:

- 0.7 mL of air + 0.7 mL of air (labeled Air, used for standardization);
- 0.7 mL of methanol + 0.7 mL of air (labeled Met);
- 0.7 mL of air + 0.7 mL of ethanol (labeled Eta);
- 0.7 mL of methanol + 0.7 mL of ethanol (labeled MaE).

**Figure 13:**
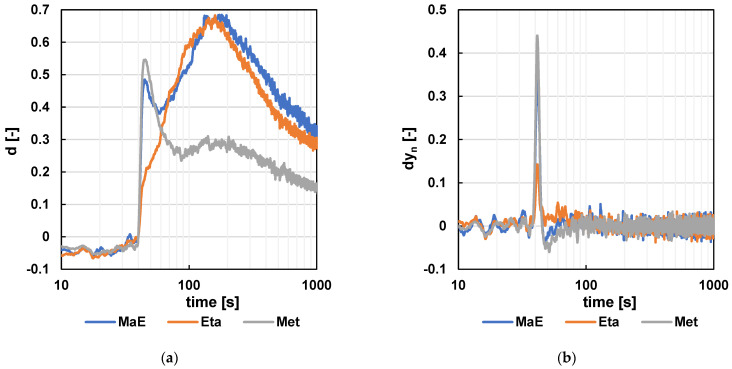
The results for the CO signal. (**a**) The resulting signal after standardization. (**b**) The resulting signal after standardization and difference calculation (m = 11).

**Figure 14:**
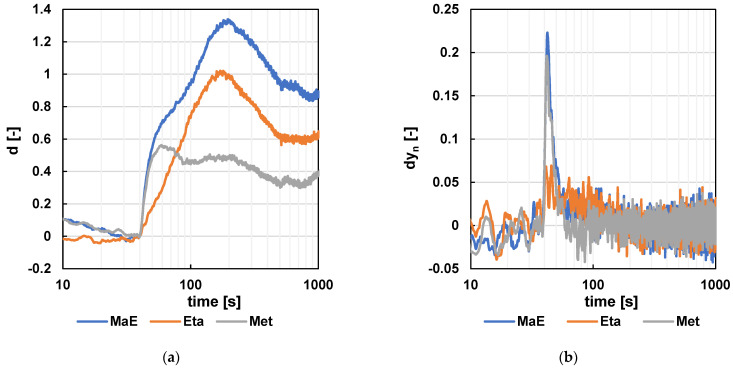
The results for the NH_3_ signal. (**a**) The resulting signal after standardization. (**b**) The resulting signal after standardization and difference calculation (m = 11).

For the resulting raw data, a pre-calculation consisting of noise elimination using the NO_2_ sensor signal and a centered moving average with parameter m = 11 was performed. The subsequent mathematical operations were consistent with the previous versions of the capillary. The signals were aligned to the same initial level, and the signal was standardized relative to the ratio of air as carrier gas to sample batch gas. Next, the difference in values (dy_n_ = x_n+5_ − x_n−5_; m = 11) was calculated to indicate the signal direction at the observed time of sample n. The resulting characteristics are shown in Figure 13 and Figure 14.

**Figure 15:**
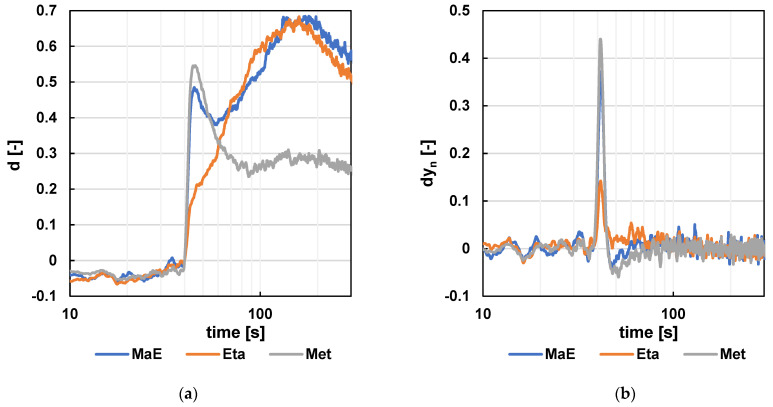
The results for the CO signal—detailed view. (**a**) The resulting signal after standardization. (**b**) The resulting signal after standardization and difference calculation (m = 11).

**Figure 16:**
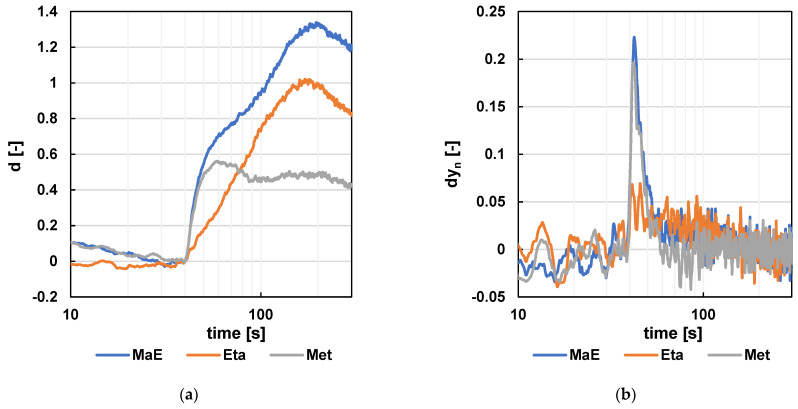
The results for the NH_3_ signal—detailed view. (**a**) The resulting signal after standardization. (**b**) The resulting signal after standardization and difference calculation (m = 11).

As seen in Figure 15 and Figure 16 (detailed view), the experimental setup successfully detected methanol, ethanol, and their 1:1 mixture. In Figure 15a, the methanol peak appears at approximately 45 s on the gray curve, while the ethanol peak is visible at 160 s on the orange curve. The blue curve, representing the methanol–ethanol mixture, shows both peaks, demonstrating distinct retention times for the two compounds, enabling their differentiation. For the NH3 sensor, the delay is even greater, causing the peaks to appear later: the methanol peak at approximately 58 s and the ethanol peak at 180 s (see Figure 16a). This delay also results in much broader peaks for the mixtures, making the methanol peak of the methanol/ethanol mixture indistinguishable.

To better differentiate the mixtures, the time differentiation of the signal was further calculated, as shown in part (b) of each figure. The inflection points of the individual samples are visible in these curves. For instance, although the methanol peak is not apparent in the ethanol/methanol mixture in Figure 16a, its inflection point occurs at the same time and with almost the same height in both the methanol–air mixture and the methanol–ethanol mixture samples.

Given the simplicity of the device, this finding could be the key information for its further applications. Methanol determination is particularly important in alcoholic beverage manufacturing to ensure the safety of consumers—its presence in food, especially alcoholic beverages, poses a real risk in developing countries. One of the many cases is the methanol case recorded in the Czech Republic during the period 2012–2013 [25]. Methanol is a highly toxic substance that, upon exposure, can cause serious health problems such as conjunctivitis, inflammation of the nasal mucosa, nerve damage, and, in severe cases, blindness [26].

A number of methods for the detection of methanol are either costly or time consuming. Examples include the determination of methoxy—and ethoxy—groups according to Willstätter and Utzinger [27]. The rapid detection of methanol is possible using, for example, a Raman spectrometer [28]. The downside of this device, however, is the very high initial cost, ranging from USD 20,000 to 50,000 [29]. The problem of ethanol and methanol detection is also addressed by Seyyed et al., who constructed a very accurate nanosensor for sensing the vapors of these substances, providing a reliable and cheaper diagnostic and monitoring device [30]. An interesting approach was also described by Angulo Barrios Carlos using a simple optical sensor based on conventional Scotch tape for the analysis of ethanol and methanol mixtures. In this case, the sensing signal is based on the change in optical power transmitted by the tape resulting from the vapor sorption reaction of the adhesive material. The sensor showed detection limits of 8.8% *v*/*v* ethanol and 17.6% *v*/*v* methanol [31].

One of the next challenges for the proposed experimental setup is, therefore, the assessment of the potential limits of the device. First, different ratios of ethanol and methanol must be tested to see if peaks for both substances are still detectable in the spectra. The subsequent step would be an attempt to quantify both substances—as mentioned by Zvonková et al. [16], the first efforts for quantitative analysis of ethanol using chemiresistive sensors and 3D printed capillary have been recorded. Results obtained from the aforementioned experiments could confirm the applicability of the device in alcoholic beverage manufacturing for both routine inspections and emergency inspections to prevent, e.g., methanol poisoning. The commercial alcoholic beverages in all conducted experiments [15,16] were chosen mostly because of their simple composition—the aim was to first evaluate the sensor’s response capabilities within the field of alcoholic beverages. If the results obtained from future experiments remain reliable within diverse ratios of ethanol and methanol, commercial samples containing more components should be introduced to see the system’s response to complex mixtures.

However, with the device’s applicability, an easy-to-use approach the and portability of the device must also be addressed. In order to be truly feasible in practical applications, the next steps of the research must also focus on the miniaturization of the device and assembling its components into a more compact arrangement.

## 5. Conclusions

An optimization of a system with a 3D printed PLA block containing a capillary, a mixing chamber, and a measuring chamber with an MiCS-6814 sensor was performed. The optimization distributed the sensor output signal in the time domain so that it was possible to distinguish the peak for the two most common alcohols, ethanol and methanol. The paper further describes some optimization types and their possibilities.

This paper presents an interesting advancement in the production of a low-cost gas separation device made of sustainable materials such as PLA, paper, and metal, which was applied in this experiment to distinguish between methanol and ethanol in food samples.

By improving the design of the device and extending its pre-separation column for chemiresistive sensors, used in a previous article, we were able to achieve better resolution and accuracy in the detection of volatile organics such as methanol and ethanol. This minor change contributed to the improved sensor performance, increased separation, and increased the versatility of the device for wider applications. At the same time, low production costs were maintained.

The possibility to differentiate between methanol and ethanol in food matrices shows the potential of the device for further use in food safety testing and quality control. These seemingly small advances represent an important step toward the development of a compact, portable, 3D printed chromatograph, for real-time monitoring and analysis in various industries.

## Figures and Tables

**Table 1:** Carrier gas flow rate table for different system configurations.

Transmission	Voltage on Motor/Time to Empty Syringe Volume	Number and Size of Syringe	Carrier Gas Flow Rate
Quick	12 V/(Not determined)	2 × 165 mL	Motor overload
	6 V/240 s	2 × 165 mL	1.375 mL/s
Slow	12 V/775 s (cca 3100 samples)	2 × 165 mL	0.4258 mL/s
	12 V/775 s (cca 3100 samples)	1 × 165 mL	0.2129 mL/s
	5 V/2525 s (cca 10,100 samples)	2 × 165 mL	0.1307 mL/s
	5 V/2525 s (cca 10,100 samples)	1 × 165 mL	0.0653 mL/s
	5 V/1800 s (cca 7200 samples)	2 × 60 mL	0.0666 mL/s
	5 V/1800 s (cca 7200 samples)	1 × 60 mL	0.0333 mL/s
	5 V/1 357.5 s (cca 5430 samples)	2 × 24 mL	0.0354 mL/s
	5 V/1 357.5 s (cca 5430 samples)	1 × 24 mL	0.0177 mL/s

## Data Availability

New research data were presented in this contribution.
